# Mitochondrial integrity in neurodegeneration

**DOI:** 10.1111/cns.13105

**Published:** 2019-02-11

**Authors:** Katrina Cowan, Oleg Anichtchik, Shouqing Luo

**Affiliations:** ^1^ Peninsula Schools of Medicine and Dentistry, Institute of Translational and Stratified Medicine University of Plymouth Plymouth UK

**Keywords:** cytotoxicity, mitochondrion, mitophagy, neurodegeneration

## Abstract

The mitochondrion is a unique organelle with a diverse range of functions. Mitochondrial dysfunction is a key pathological process in several neurodegenerative diseases. Mitochondria are mostly important for energy production; however, they also have roles in Ca^2+^ homeostasis, ROS production, and apoptosis. There are two major systems in place, which regulate mitochondrial integrity, mitochondrial dynamics, and mitophagy. These two processes remove damaged mitochondria from cells and protect the functional mitochondrial population. These quality control systems often become dysfunctional during neurodegenerative diseases, such as Parkinson's and Alzheimer's disease, causing mitochondrial dysfunction and severe neurological symptoms.

## 1 INTRODUCTION

The mitochondrion is a complex intracellular organelle. Its high abundance in cells gives a strong indicator as to its importance in cellular function. Their number and shape differ according to the cell type.^1^ Research so far has revealed that mitochondria have multiple important roles, including ATP production,^2^ Ca^2+ ^homeostasis,^3^, ^4^ and reactive oxygen species (ROS) production.^5^, ^6^

The mitochondrion has a unique evolutionary background, where it is thought that they originated from α‐proteobacteria.^7^ Evidence of this resides in the presence of mitochondrial DNA. Unlike genomic DNA, mitochondrial DNA is circular in form and therefore has similarities to bacterial DNA in structure. While mitochondrial DNA does code for some proteins important for mitochondrial function, the majority of mitochondrial structural proteins are encoded by the cell nucleus, where the proteins are imported and sorted into the mitochondria.^8^ In addition to structure, mitochondrial DNA differs from genomic DNA in the copy number, where often mitochondria can contain many more copies of DNA. Mitochondrial DNA is inherited predominantly through the maternal line,^9^ and therefore, any mitochondrial DNA mutations will pass from the mother to her children.

Due to the diverse and crucial role of the mitochondrion within the cell, the mitochondrial integrity is important to cell survival. There are a large number of diseases where mitochondria, either directly or indirectly, become dysfunctional. This review aims to describe the processes for mitochondrial function, the mitochondrial pathways that are initiated during mitochondrial dysfunction, and how this relates to neurodegenerative diseases.

## 2 FUNDAMENTALS OF MITOCHONDRIAL BIOLOGY

### 2.1 Mitochondrion structure

Healthy mitochondria are required for sufficient ATP production and cellular function. The mitochondrion, consisting of a jelly‐like matrix, is a double‐membrane organelle found in multiple copies within each cell. They are encompassed by the inner mitochondrial membrane (IMM), a highly folded membrane. Beyond the IMM is the intermembrane space (IMS), which is between the IMM and the outer mitochondrial membrane (OMM).^10^

The matrix is a key area for respiration, as within this space, both the tricarboxylic acid (TCA) cycle and the breakdown of pyruvate into adenine triphosphate (ATP) take place. This process leads to the production of H^+ ^ions which are necessary for the final step of mitochondrial respiration. Two important protein produced within the matrix are FADH_2_ and NADH. These molecules are important substrates for mitochondrial respiration. NADH is used as substrate to reduce ubiquinone to ubiquinol in complex II of the electron transport chain (ETC).^11^ NADH transfers electrons to complex I, the NADH Q reductase, which causes NADH to be reduced into NAD^+^ and releases H^+^ ions into the matrix. Embedded within the IMM are several more proteins that make up the ETC Complex II, III, IV, and V are also found in the IMM, and together they form the ETC One important component of the ETC is cytochrome *C*, which plays an important role in both ATP production and cell apoptosis.^12^ This will be discussed in further detail later on. The OMM is the second mitochondrial membrane, separating the IMS from the cytosol. There are multiple proteins found on the OMM, with a diverse range of functions, such as receptors for autophagy molecules, and membrane proteins involved in mitochondrial division and fusion.

**Figure 1:**
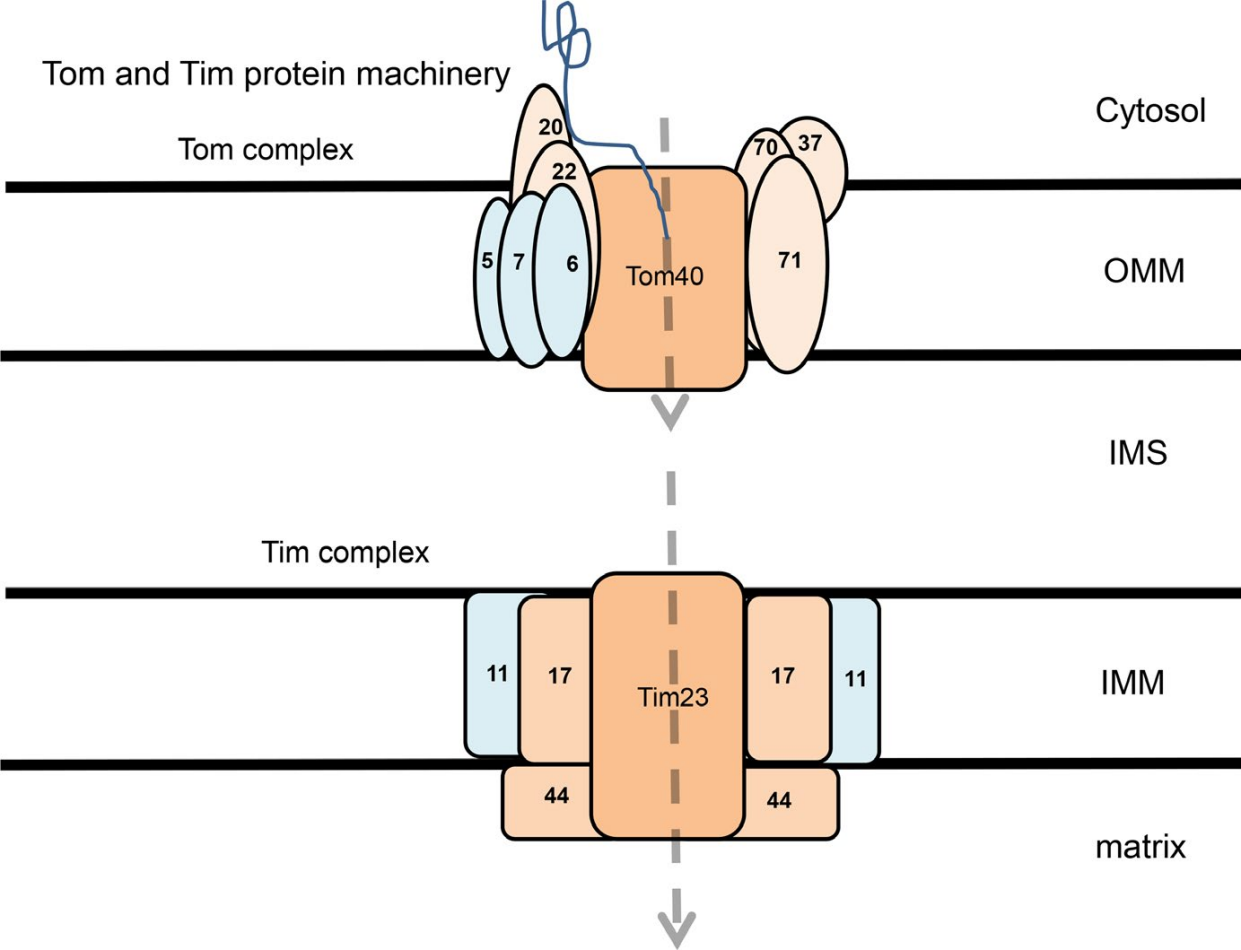
The Tim and Tom complexes. The key pore proteins are Tom40 and Tim23, for the OMM and the IMM, respectively. In the Tom complex, smaller subunits Tom5, Tom6, and Tom7 are within the OMM. Larger subunits Tom20, Tom22, Tom37, Tom70, and Tom71 are receptor proteins, which bind to proteins and guide them into the IMS. The main Tim complex is comprised of additional subunits Tim11, Tim17, and Tim44. Proteins enter to the matrix via the Tom and Tim complexes. Proteins such as alpha‐synuclein bind to the receptor Tom20, which then guides them into the Tom40 pore (blue line)

Both the IMM and OMM contain mitochondrial translocase proteins, Tim, and Tom proteins, respectively. These proteins have an important role in the import of mitochondrial structure proteins. Both the Tim and Tom proteins consist of multiple subunits, which form a complex spanning the mitochondrial membrane. The major subunits which form the pore in the IMM and OMM, Tim23 and Tom40, respectively, allow passage through the double membrane into the matrix. In the IMM, five other different subunits have been identified: Tim17, Tim23, Tim44, Tim11 (these four subunits have been found to be closely associated with each other), and Tim22, which is important for cell survival. In the OMM, the Tom complex consists of nine subunits: Tom5, Tom6, Tom7, Tom20, Tom22, Tom37, Tom 70, Tom72, and Tom40. The Tom complex draws proteins into the central pore, Tom40, via the receptor subunits, Tom20, 22, 37, 70 and 72, and through into IMS (Figure 1). The Tom and Tim machinery is crucial for mitochondrial structure as mitochondria import the vast majority of their structure proteins.^13^

### 2.2 Mitochondrial membrane potential

**Figure 2:**
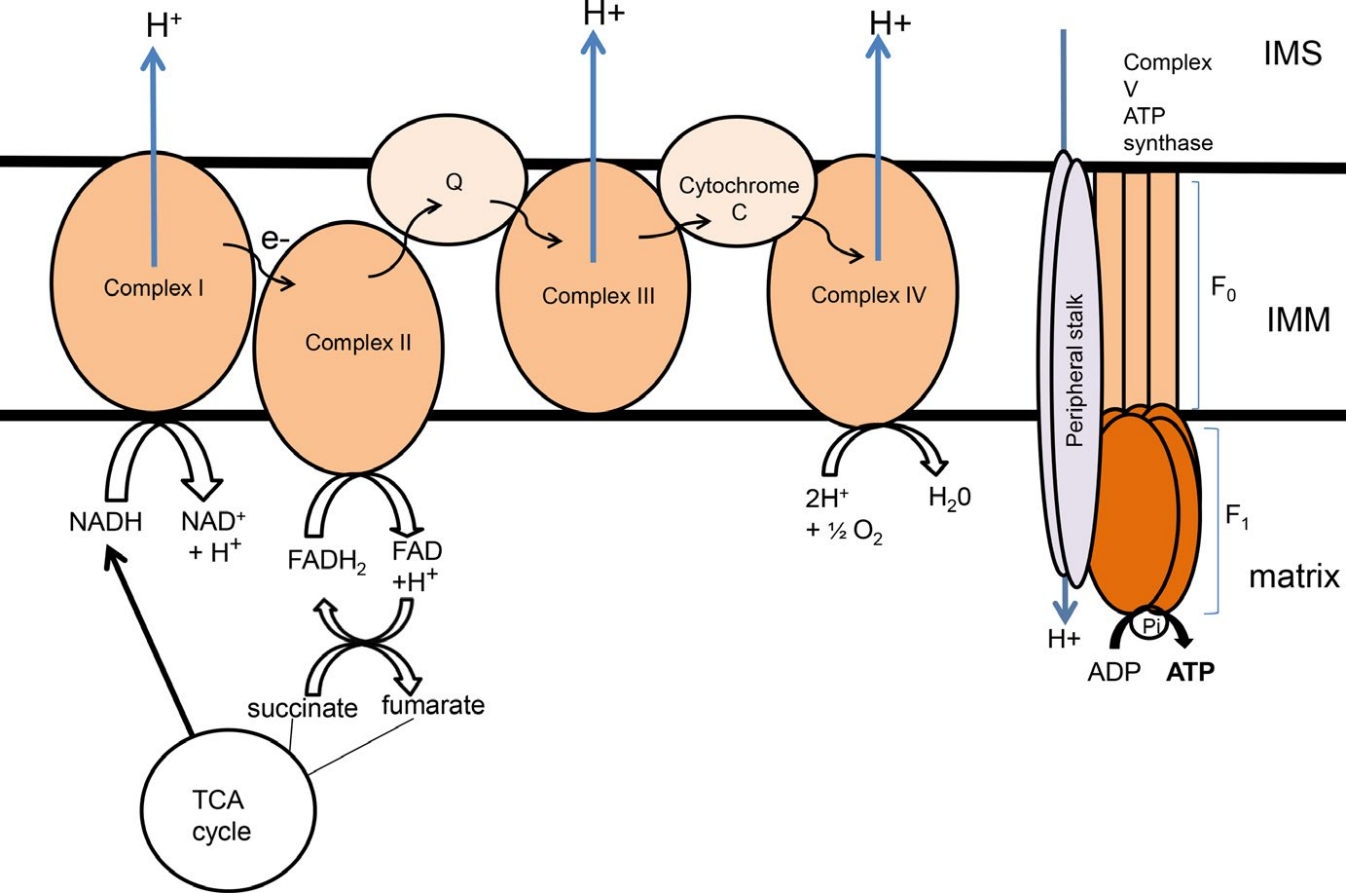
Structure of the ETC The ETC is comprised of five main complexes which span the IMM. In complex I, NADH, which is produced in the TCA cycle, is reduced to NAD^+^ and H^+ ^ions. The H^+^ ions are pumped out into the IMS and the electrons (e−) are passed onto complex II, which catalyzes the reduction in FADH_2_ to FAD. Complex II is also a part of the TCA cycle, and FAD can also be oxidized to form FADH_2_, which in turn reduces succinate to form fumarate in the TCA cycle. Electrons are passed through the coenzyme Q, reducing ubiquinone into ubiquinol, and then into complex III. Cytochrome *C* allows the flow of electrons from complex III to complex IV. At complex IV, H^+^ ions in the matrix react with O_2_ molecules to form H_2_O. In addition H^+^ ions are also pumped out into the IMS. At complex V, also known as ATP synthase, H^+^ ion, which have accumulated in the IMS, then pass down the concentration gradient back into the matrix. This H^+^ ion flow causes the F_0_F_1_ ATPase to rotate, which catalyzes the phosphorylation of ADP, generating ATP molecules into the matrix

The mitochondrial membrane potential (sometimes denoted as Δψm) is generated by protons pumped into the inner membrane space (IMS) of the mitochondrion. This occurs in the final step during oxidative phosphorylation, which also results in ATP production. Electrons are passed along the ETC, initiated by the transference from substrates such as NADH, from the matrix to the ETC^14^ Electron flows laterally along the IMM then reaches the terminal ETC complex, F_0_F_1_‐ATP synthase enzyme, or otherwise known as complex V of the ETC, situated on the IMM^15^, ^16^ (Figure 2).

The generation of ATP from ADP is catalyzed by the F_0_F_1_ ATP synthase enzyme. This reaction requires the movement of protons through the IMM, from the IMS into the matrix. The F_0_F_1_ ATP synthase enzyme consists of two complexes, the F_0_ and the F_1_. These proteins act as motors, which are able to twist and change conformation. They are connected by central stalks, which allow them to affect each other's movement and activity. The F_1_ component is found protruding outside the IMM, into the matrix of the mitochondrion. In contrast, the F_0_ component is found embedded in the IMM. Studies have shown that the F_0_ component is vital for the movement of protons across the mitochondrial membrane and for the production of the mitochondrial membrane potential. The F_1_ component, however, is important for the generation of ATP, as well as the hydrolysis of ATP into ADP. Depending on the enzymatic reaction, whether it might be ATP production or hydrolysis, the rotation of the F_1_ motor is changed which alters the spin of the unit.^17^ When the H^+^ ions cross from the IMS into the matrix, ADP is converted to ATP, which is used as a form of energy by the cell. This generates an electrochemical gradient across the inner membrane (Figure 2). Measuring this electrochemical gradient has been proposed to be a relative indicator of the generation of ATP.^16^ In addition to ATP production, mitochondrial membrane potential is also directly involved in import of important ions and mitochondrial structural proteins. The majority of mitochondrial proteins are imported into the mitochondrion as precursor proteins. These precursor proteins contain either mitochondrial targeting sequences, which direct the protein to the correct apartment, or they contain N‐terminal presequences that allow them to pass through the OMM and IMM, after which they are cleaved. The electrostatic charge across the mitochondrial membrane, with a negative intracellular charge, allows the import of positively charged precursor proteins and cations such as Ca^2+^ or Fe^2+^. The precursor of cytochrome *b*_2_, for example, which is found in the IMM, is one such protein that relies on the mitochondrial membrane potential for its translocation into the mitochondria. It was found that cytochrome *b*_2_ contained a positively charged N‐terminal sequence, which drove the movement of the protein toward the negatively charged IMM, first into the matrix and then to be sorted into the IMS. When the mitochondrial membrane potential is abolished, the import of cytochrome *b*_2_ into the mitochondrion is inhibited, suggesting that the translocation of cytochrome *b*_2_ into the matrix is dependent on the mitochondria membrane potential generated across the IMM.^18^, ^19^, ^20^

So far, there are two proteins which are thought to be involved in the maintenance of mitochondrial membrane potential; these being ATPase inhibitory factor 1 (IF1) and adenine nucleotide translocase (ANT). The first modulator, IF1, is a nuclear‐encoded protein. It works as an endogenous inhibitor of ATP synthase, by binding directly to the F_0_F_1_ ATPase.^21^ ANT is an ADP/ATP carrier where it catalyzes the conversion of ADP to ATP in the IMM and is closely involved in oxidative phosphorylation. In stressful conditions where mitochondrial function is impaired, the ADP/ATP carrier attempts to maintain the mitochondrial potential, by prioritizing the membrane potential over ATP production, and causes the carrier to reverse the enzymatic reaction, so that ATP is converted into ADP, exporting it into the cytoplasm.^22^

### 2.3 Mitochondrial permeability transition pore

The mitochondrion contains certain voltage‐dependent channels within the mitochondrial membrane. This pore, when open, allows certain elements to flow from the mitochondrion, thus altering and controlling the permeability of the mitochondrial membrane. This can therefore allow the transport of proteins, which otherwise would be impermeable to the IMM. When the permeability pore is open, small proteins of up to 1.5 kDa, which otherwise are impermeable, can pass through the IMM.^23^

The transition pores can be either in a reversible open or closed state. The probability of the high conductive pore opening can be modulated by both the mitochondrial membrane potential and the pH gradient. In fact, it had been suggested that part of the ATP synthase, the machinery which generates the mitochondrial membrane potential, forms part of the mitochondrial permeability transition pore (MPTP).^24^ The permeability of the pore is intrinsically related to changes in Ca^2+^ ion homeostasis, which is a strong and essential trigger for MPTP opening. In this regard, the mitochondrial calcium uniporter (MCU), a channel which allows calcium transport into the matrix, has a direct effect on the MPTP.^25^

The MPTP has caught the interest of scientists for many years. However, despite this, there has been little conclusive evidence of its structure. There have many studies with the aim to determine the proteins involved in the MPTP and over the years different proteins have been proposed, including adenine nucleotide translocase (ANT) and the voltage‐dependent anion channel (VDAC). ANT was proposed many years ago, by the scientists Hunter and Haworth, as it had been shown to be sensitive to certain ligands which can modulate MPTP opening.^26^ However, firm confirmation of its direct involvement in MPTP function has not yet been made, so it is unclear how important the protein is in the MPTP. VDAC, a protein found in the OMM, is an ion channel pore that changes it open or closed state in the response to changes in the mitochondrial membrane potential. VDAC is thought to play an important role in membrane permeability, but it had also been found to be equally important in apoptosis. The proteins Bax/Bak interact with VDAC and cause the pore to open more readily, which would allow cytochrome *C* to be exported into the cytosol.^27^ However, the protein cyclophilin D (CypD) is generally accepted as a protein found in the MPTP.^28^ The importance of CypD was discovered mainly through the effect of the drug cyclosporine A (CsA), which had an inhibiting effect on CypD.^25^ The addition of CsA was found to desensitize the MPTP to Ca^2+^ ions, preventing MPTP opening. The precise function of CypD within the MPTP is not clear however. It has been suggested that CypD may regulate the opening of the MPTP; the mechanisms by which this happens however are not well known either.

### 2.4 Cytochrome *C*

Mitochondrial metabolism is a key mitochondria process and is heavily regulated through redox reactions. One of the key proteins involved in these redox reactions is cytochrome *C*. Cytochrome *C* is normally situated within the IMS, where it has an important role in mitochondrial respiration. The size restriction, of 1.5 kDa, prevents larger proteins, such as cytochrome *C *from passing through the MPTP. The release of cytochrome *C* occurs when the OMM is disrupted, which releases cytochrome *C* out of the IMS into the cytoplasm.^29^, ^30^ Cytochrome *C* has a diverse range of cellular functions. In addition to apoptosis, it has been found to be heavily involved in the mitochondrial ETC, where it acts as an electron carrier between complex III and complex IV. Complex III within the ETC consists of a bc1 complex. The bc1 complex transfers an electron to complex IV. Cytochrome *C* augments this transfer to complex IV, by transferring the electron onto cytochrome oxidase in complex IV. After four such electron transfers, oxygen is then converted to water and two electrons are released from the matrix into the IMS.^31^

In addition, cytochrome *C* is able to reduce the effects of ROS, by acting as a ROS scavenger and removing electrons from oxygen species. ROS includes molecules such as superoxide (${\text{O}}_{2}^{\cdot -}$) and hydroxyl radical (·OH), both of which are classed as free radicals, and hydrogen peroxide (H_2_O_2_). These ROS molecules contain oxygen, which have free radicals and are highly reactive and toxic, even to DNA. These ROS species are formed from the oxygen molecules produced from the electron transport chain, especially in complex I and III.^32^ Complex III, a multisubunit complex also named the cytochrome bc1 complex, is found within the IMM, is able to generate ROS via the Qo site within complex III, which then causes ROS to be released into the IMS.^33^ Although complex I and complex III are the major sources of ROS production in the mitochondrion, complex II has also been found to produce ROS in certain conditions.^34^ Complex II catalyzes the conversion of succinate to fumarate, but when there are low levels of succinate, or if complex I or III are inhibited, complex II can produce superoxide molecules or hydrogen peroxide in the matrix. Due to its importance in ROS production and apoptosis, cytochrome *C* is closely regulated. Cytochrome *C* can be modulated by ATP and phosphorylation. ATP can bind to directly to cytochrome *C*, which inhibits its activity.^35^ There have been several phosphorylation sites identified so far, including Tyr97, Try48, and Thr28, when these sites are phosphorylated, it reduces the electron flow across the ETC and prevents ROS production and apoptosis.^36^

## 3 MITOCHONDRION‐RELATED CYTOTOXICITY

### 3.1 Mitochondrial‐dependent apoptotic death

One of the key roles of the mitochondrion is the induction of apoptosis. Under pathological conditions, cell death signaling molecules are released from the mitochondrion into the cytoplasm, triggering apoptosis of the cell. This occurs through the permeabilization of the outer mitochondrial membrane, which enables these molecules to leave the IMS into the cytoplasm.^37^ Mitochondrion‐dependent apoptosis is regulated by the Bcl‐2 family proteins. The Bcl‐2 family proteins are comprised of three groups of proteins: Bax/Bak proteins, Bcl‐2‐like proteins, and Bcl‐2 homology 3 (BH3)‐only proteins.^38^

**Figure 3:**
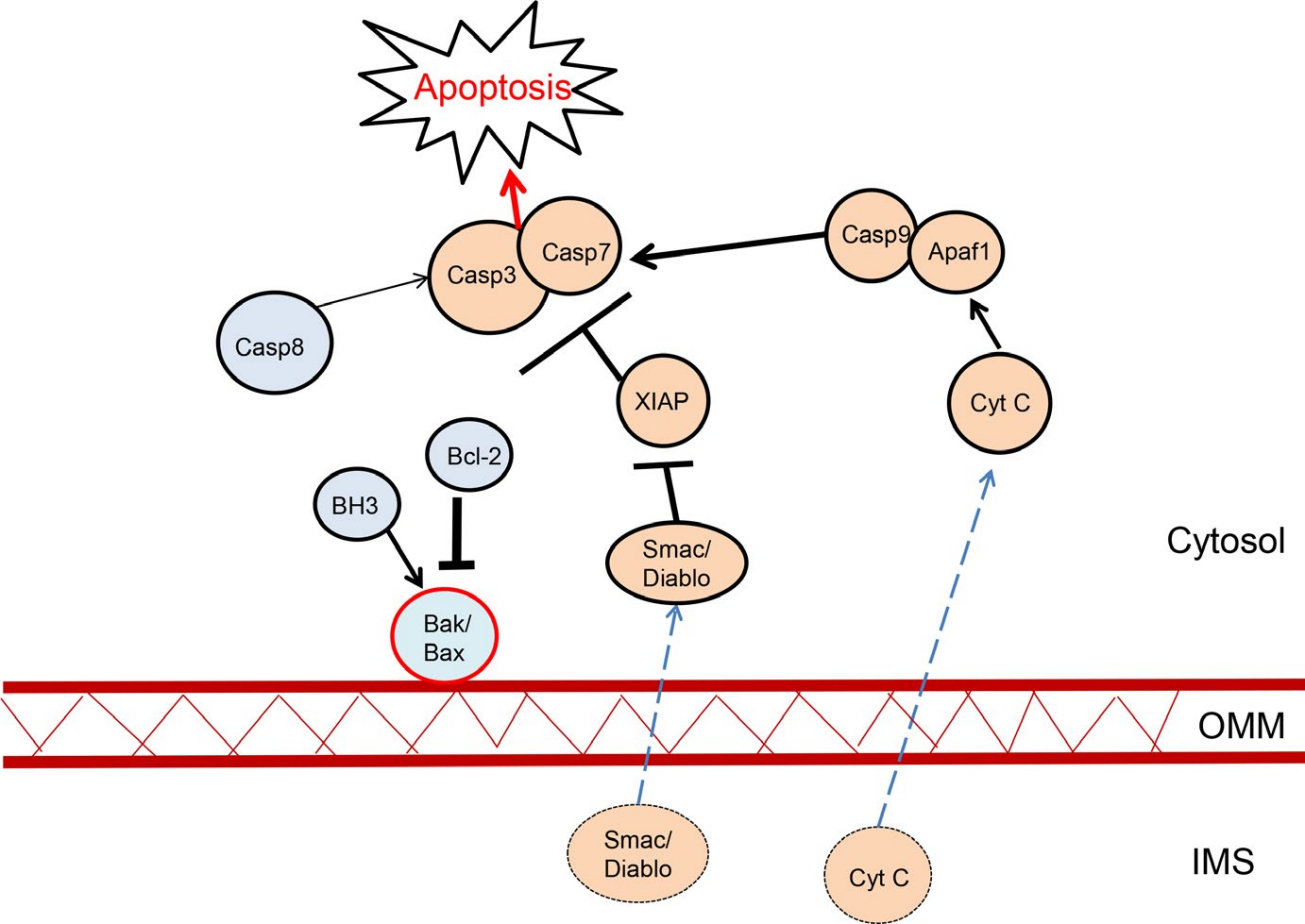
Mitochondrion‐dependent apoptosis. When the mitochondrion is damaged, BH3‐only proteins activate Bak and/or Bax, causing permeabilization of the OMM (red line representing the permeabilization of the OMM). Bcl‐2 proteins including Bcl‐2, Bcl‐x, and MCL inhibit Bak/Bax activity. Once the OMM is permeabilized, several IMS‐localized proteins, such as cytochrome *C*, transport out of the mitochondrial in the cytosol (dotted line represents the movement of cytochrome *C*). Cytochrome *C* activates Apaf‐1, which in turn binds to caspase‐9, to form a complex, the apoptosome. This complex then activates caspase‐3 and caspase‐7, and leads to apoptosis. In addition to cytochrome *C*, the IMS‐localized protein Smac/Diablo enters the cytosol and inhibits XIAP, which is a caspase‐3 and caspase‐7 inhibitor. This leads to higher levels of caspase‐3 and caspase‐7 in the cytosol

Bax/Bak proteins are directly involved in apoptosis, where they form a channel within the OMM, causing OMM permeabilization.^39^ Once this happens, mitochondrion‐dependent apoptotic death is determined. In addition to the Bax/Bak protein group, the BH3‐only protein group was found to be indirectly involved in mitochondrial permeabilization. The BH3‐only proteins, such as truncated Bid (tBid), have been identified as such factors. tBid is a BH3 interacting domain death agonist and can stimulate Bax/Bak activation and translocation to the OMM. tBid, once activated by the key pro‐apoptotic protein caspase‐8, activates Bak/Bax which causes permeabilization of the OMM.^38^, ^40^ tBid, and other BH3‐only proteins, such as Bim, PUMA and Noxa, can also bind to Bcl‐2 proteins and inhibit their activity.^38^ Bcl‐2 proteins are able to bind to Bax/Bak, reducing OMM permeabilization caused by the latters. When tBid, Bim, PUMA, or Noxa binds to Bcl‐2 proteins, it allows Bax/Bak to become activated and increases OMM permeabilization (Figure 3).

A key signaling molecule during apoptosis is the caspase protein, which is released into the cytoplasm. Caspases are cysteine aspartate proteases. Its activation can be induced by several different stimuli, such as DNA damage, tumorigenesis, and oxidative stress.^41^ After OMM permeabilization, certain proteins are released from the mitochondrion into cytosol, which then in turn activate caspases. These include the proteins Smac, Omi, and cytochrome *C*, which can all be found in the IMS. Smac and Omi both help to initiate caspase activity by binding and preventing the actions of a caspase inhibitor XIAP.^42^

While more commonly known as an important ETC protein, cytochrome *C* is a particularly important IMS protein for caspase activation. Cytochrome *C* binds to the caspase adaptor molecule Apaf‐1, altering its conformation. Apaf‐1 recruits caspase‐9, together forming a large collective complex, known as the apoptosome. This recruitment occurs through the exposure of the caspase activation and recruitment domains (CARD) in Apaf‐1*.* 43 Through caspase‐9 dimerization, the apoptosome is activated, which then cleaves and activates, caspase‐3 and caspase‐7*,* causing severe cell death^42^ (Figure 3)*.*

### 3.2 Mitochondrial‐derived neuroinflammation

In the same way, that cells exhibit an inflammatory response to invading pathogens, mitochondrial damaged‐associated molecular pattern (DAMPs) is a process by which a damaged mitochondrion releases signaling molecules into the cytoplasm, in response to stress. It has been suggested that this process may be involved in many diseases, including heart disease, sepsis,^44^ and neurodegeneration. There is an intricate relationship between mitochondrial DAMPs, which are also known as alarmins, and neuroinflammation. Mitochondria release signaling molecules which are recognized by PRRs (pattern recognition receptor), such as the toll‐like receptors (TLRs) or the NOD‐like receptors (NLR). NLR3, one such member of the NLR family, is normally found in the endoplasmic reticulum. When the agonist of NLR3 is bound, it causes the receptor to translocate to the mitochondrion to oligomerize and this recruits the protein ASC, apoptosis‐associated speck‐like protein with a caspase activation and recruitment domain (CARD). This complex then binds to procaspase‐1 which altogether forms the NLRP3 inflammasome.^45^, ^46^ There have been several molecules that were specifically identified as DAMP molecules; these include ATP, cytochrome *C*, mtDNA,^47^ and mitochondrial transcription factor A (TFAM).^48^ These DAMP molecules are recognized by the PRRs, which initiates the cascade of pro‐inflammatory pathways, as well as initiating astrogliosis and activating microglia.

Mitochondrial dysfunction, which results in the generation of DAMP molecules response, leads to the recruitment of key pro‐inflammatory molecules such as interleukin‐1β (IL‐1β) and cytokines such as TNFα. These cytokines however can themselves affect mitochondrial function, with TNFα capable of impairing the function of mitochondrial ETC components, reducing ATP production, increasing ROS production, and depolarization of the mitochondrial membrane potential.^46^ Increased ROS can then have further effects by initiating activation of the nuclear factor NF_k_B, a key regulator of cytokine production, DNA transcription and pro‐inflammatory signaling. The stimulation of neuroinflammation can therefore also have a detrimental effect on mitochondrial function itself, thus creating a vicious cycle.

## 4 QUALITY CONTROL IN THE MITOCHONDRION

### 4.1 Mitochondrial dynamics

Mitochondria have the ability to bind to other mitochondria, as well as to divide into two different mitochondria. These processes are collectively known as mitochondrial dynamics. This process allows the mitochondria to change morphologically, and increase or decrease the mitochondria number within the cell. The process is mediated by a group of proteins, the dynamin family, which all contain a GTPase domain. In addition to sharing this GTPase domain, they also share other domains: a middle domain, a variable domain, and a GTPase effector domain. All members of the family are able to bind to membranes, which occurs through activation of the variable domain.^49^

Mitochondrial fusion is the process by which two mitochondria fuse together to form one mitochondrion. This occurs by the binding of membrane‐bound proteins on the outer membranes of the mitochondria. The fusion of the inner mitochondrial membrane often then follows to form one mitochondrion, with the newly combined mitochondrion containing the contents of both mitochondria, such as the matrix and nutrients. This allows mitochondria to reduce any potential cell‐damaging molecules that may be in a segregated mitochondrion, ensuring mitochondrial survival and normal function. The process of mitochondrial fusion causes mitochondria to elongate, forming rod‐shaped mitochondria. The main proteins involved in mitochondrial fusion are Opa1, mitofusin 1 (Mfn1), and mitofusin 2 (Mfn2). During fusion, Mfn1 and Mfn2, found on the OMM, bind to the same receptors on the neighboring mitochondria, which bring the two mitochondria together. The two OMMs fuse together creating one continuous membrane.^50^

Mitochondrial fission, on the other hand, is regulated almost exclusively by the protein drp1 (dynamin‐related protein 1). The precise mechanism of drp1 fission is not yet understood; however, it is known that drp1 binds to receptors on the OMM and oligomerizes to form a ring‐like structure around the mitochondria. The mitochondrion is then divided into two daughter mitochondria, via GTPase hydrolysis. Drp1 proteins are recruited from the cytosol and bind to specific receptors on the OMM. The proteins MiD49, MiD51, Fis1, and Mff have recently all been identified as drp1 receptors on the OMM. MiD49 and MiD51, or mitochondrial dynamics proteins of 49 and 51 kDa, are able to mediate drp1 fission by forming foci at the OMM and recruiting drp1 to the mitochondria. Knockdown of the MiD receptor prevented drp1 recruitment, leading to increased mitochondrial fusion. However, although they are able to recruit drp1, it was found that the MiD receptors dissociate from the OMM during GTPase hydrolysis. It was discovered that overexpressing the MiD receptors can prevent mitochondrial fission.^51^, ^52^ Fis1 is a protein first found to interact with the drp1 orthologue, Dnm1, in yeast,^53^ however, it was discovered that the interaction was not directly between drp1 and Dnm1, but rather through two adaptor molecules, Mdv1 and Caf4.^54^, ^55^ These adaptor molecules are not found in mammals, so the role of Fis1 in mammalian mitochondrial fission is not entirely clear. There has been a mixture of results, with some saying that Fis1 is not necessary for mitochondrial fission,^56^ and some studies showing Fis1 does interact and cause drp1‐dependent fission.^57^, ^58^ The receptor Mff on the other hand has been accepted the most as a mitochondrial fission receptor.^56^, ^57^, ^59^ A knockdown in Mff levels leads to a reduction in mitochondrial fission and an increase in the elongation of the mitochondria.^58^ Mitochondrial fission can help to control the mitochondrial quality by segregating damaged or unhealthy mitochondria away from the healthy mitochondria, and which would then be singled out for degradation via the mitophagy pathways.

### 4.2 Mitophagy

Mitophagy is the process in which damaged mitochondria are degraded by the autophagy system. This process is vital for removing debris and aggregating proteins from the cells, thereby relieving the cells of potential cell‐damaging proteins. Damaged mitochondria are singled out and degraded, through the activities of the proteins PTEN‐induced kinase 1 (Pink1) and Parkin.^60^

Both Pink1 and Parkin are localized near healthy mitochondria in the cytosol. Pink1, a serine/threonine kinase, is encoded by nuclear DNA and has been shown to be involved upstream of Parkin, an E3 ubiquitin ligase. The two protein work together to keep cells healthy by helping to clear any damaged mitochondria from the cell.^61^

**Figure 4:**
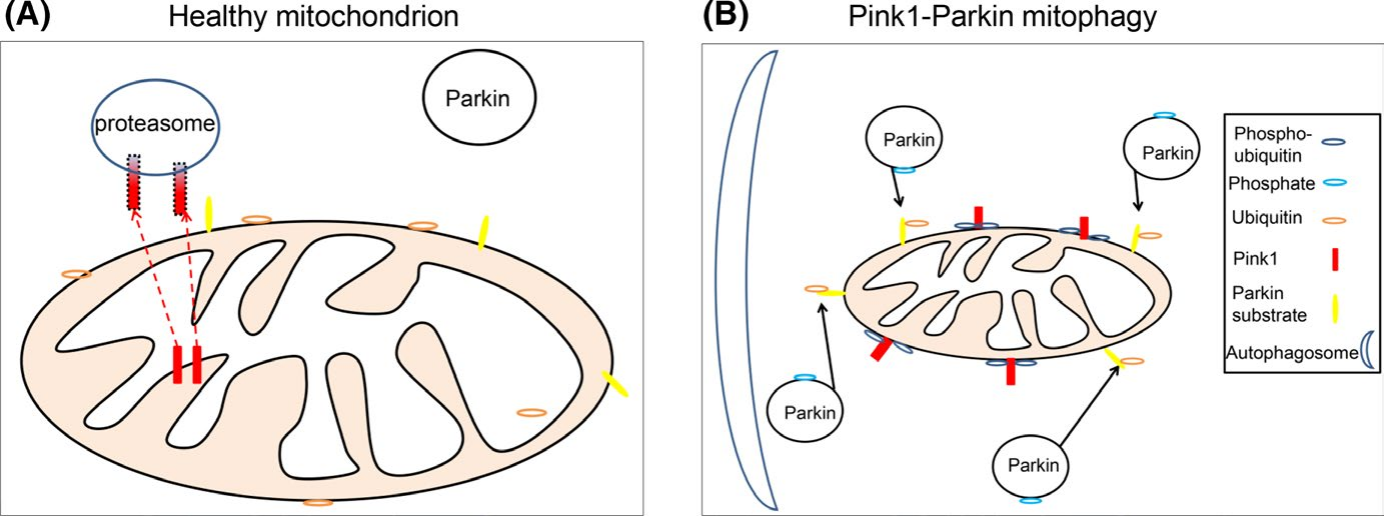
Pink1 and Parkin in mitophagy in healthy and dysfunction mitochondria. A, In normal healthy mitochondria, Pink1 is predominantly expressed in the IMM. Pink1 is then translocated to the cytosol, where Pink1 is digested by the proteasome. Pink1 does not ubiquitinate OMM proteins and the cytosolic protein Parkin, and so Parkin is not activated. B, When mitochondria are extremely damaged, the Pink1‐Parkin pathway is activated. Pink1, instead of being degraded by the proteasome, is translocated to the OMM, which leads to its accumulation on the OMM. Pink1 phosphorylates both ubiquitin on the OMM and Parkin. The E3 ligase activity of Parkin is initiated, Parkin then ubiquitinates protein substrates, such as Miro, on the OMM, and also recruits the autophagosome to the mitochondrion for mitochondrial degradation

In physiological conditions, Pink1, imported from the cytosol via mitochondrial translocases, is usually found within the IMM, where it is exported and proteolytically cleaved and degraded. Pink1 was discovered to have a mitochondrial targeting sequence (MTS) which directs the protein into the correct mitochondrial subcompartment. Pink1 is imported into the mitochondrion as a precursor, which is cleaved within the matrix by the protease MPPα/β.^62^ While it is thought that the N‐terminus of Pink1 has some involvement in mitochondrial targeting, it has also been discovered that there are cleavage sites situated within the N‐terminus which are cleaved by the MPP proteases. The import of Pink1 through the Tom complex is driven by the mitochondrial membrane potential, and once through, Pink1 then transports across the IMM via the Tim complexes. In its full form, Pink1 itself exists at 64 kDa. However, there have been reports of further cleavage of overexpressed Pink1, resulting in a smaller 52 kDa form.^63^, ^64^ This cleavage occurs when Pink1 binds to the protease PARL (presenilin‐associated rhomboid‐like), which is found within the IMM. Pink1 is then cleaved within its transmembrane domain and is then transported to the cytosol where it is degraded by cytosolic proteasome (Figure 4A).

When a mitochondrion is damaged, Pink1 levels at the OMM are increased. During mitochondrial damage, the mitochondrial membrane potential is altered and Pink1 is prevented from being able to enter through the OMM, causing Pink1 to accumulate on the OMM. Pink1 recruits Parkin, normally found in the cytosol, by phosphorylating ubiquitin found on certain protein on the OMM, which draws Parkin toward the mitochondrion. In addition to OMM surface proteins, Pink1 phosphorylates Parkin. This causes Parkin to become activated, and once activated Parkin initiates the process of degradation of the mitochondrion, where the mitochondrion is engulfed by the autophagosome and then consequently fuses with the lysosomes. Activated Parkin can then ubiquitinate other mitophagy receptors on the OMM. Both Parkin and Pink1 therefore contribute to the build‐up of phosphorylated ubiquitin and abundant accumulation of Pink1 on the OMM, which acts as an indicator to the cell that the mitochondrion is damaged and will need to be removed^65^ (Figure 4B).

## 5 MITOCHONDRIAL DYSFUNCTION IN NEURODEGENERATION

**Figure 5:**
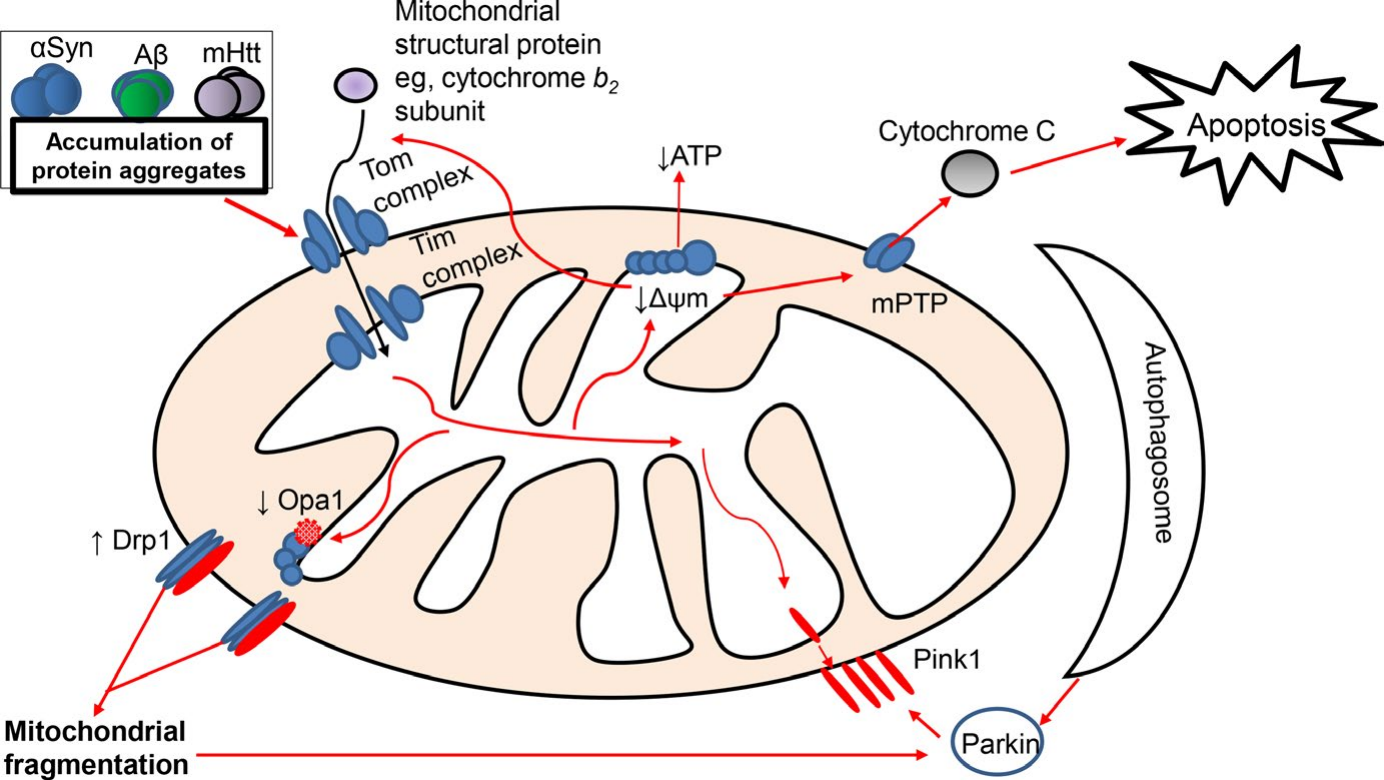
An overview of mitochondrial dysfunction in PD (asyn), AD (Aβ), and HD (mHtt). Protein aggregates formed in these three neurodegenerative diseases negatively impact mitochondrial function. Although the exact mechanisms are not entirely understood, protein aggregates can lead to a disruption in the mitochondrial membrane potential, resulting in decreased ATP production. This disruption in membrane potential can lead to the permeabilization of the OMM, by opening the mitochondrial permeability transition pore (mPTP), leading to the release of pro‐apoptotic molecules such as cytochrome *C*. The impairment of the mitochondrial membrane potential can also prevent the import of mitochondrial structural proteins, such as the subunit cytochrome *b_2_*, which are normally produced in the nucleus and are translocated into the mitochondria through the Tom and Tim complexes. Protein aggregates can also disrupt mitochondrial dynamics (Drp1 and Opa1), leading to abnormal mitochondrial number and fragmentation. When the mitochondrion is damaged beyond repair, the mitophagy process is activated, which involves the recruitment of Pink1 to the OMM, which in turn recruits Parkin from the cytosol and activates it. The activation of Parkin then leads to the formation of the autophagosome, which degrades the damaged mitochondrion, removing it from the general mitochondrial population

Research into neurodegeneration so far has consistently reported changes in mitochondrial function, such as reduced ATP production and increased mitochondrial fragmentation. These mitochondrial dysfunctions can be seen in many different neurodegenerative diseases, suggesting that the underlying pathways behind mitochondrial dysfunction are somewhat shared in all neurodegenerative diseases (Figure 5).

### 5.1 Parkinson's disease

Parkinson's disease (PD), the second most common form of neurodegenerative disease, is characterized by the accumulation of the protein alpha‐synuclein, especially in the substantia nigra. This protein, found abundantly throughout the central nervous system, accumulates and forms Lewy bodies, a defining feature which is commonly found in the postmortem examination of PD patients.^66^ The relationship between alpha‐synuclein and mitochondria has been explored in several papers. It was shown that alpha‐synuclein can bind to and be imported directly into the mitochondria, which, when oligomerized, can have an adverse effect in PD.^67^ Normal alpha‐synuclein is thought to have some beneficial role in mitochondrial function. Alpha‐synuclein, for example, is thought to bind to lipid membranes, such as the lipid membrane of the endoplasmic reticulum (ER), or more specifically the mitochondrion‐associated ER membrane, through which the ER interacts with the mitochondrion.^68^ The study shows that mutated alpha‐synuclein reduces this interaction between the ER and the mitochondrion, suggesting that alpha‐synuclein has an important role in the communication between the two organelles.

Alpha‐synuclein was found to be directly localized with mitochondria. Isolated mitochondria from PD patients were found to contain alpha‐synuclein.^69^ Alpha‐synuclein has been shown to have a strong affinity to mitochondria.^67^ This may be because alpha‐synuclein has an affinity to cardiolipin, which is found throughout the IMM.^70^ Due to this relationship between alpha‐synuclein and mitochondria, research has been directed into understanding the changes in mitochondria in PD, and other synucleinopathies. The oligomerization and aggregation of alpha‐synuclein have been shown to cause deficits in the activities of complex I, which leads to a reduction in ATP production, depolarization of the IMM, and the release of pro‐apoptotic molecules into the cytosol.^71^, ^72^

Studies into PD have also shown that alpha‐synuclein has a direct effect on mitochondria appearance, causing fragmentation of the mitochondria. Using a model of overexpressed alpha‐synuclein in dopaminergic neurons, some studies suggest that drp1 levels increase which causes increased fragmentation, cleavage of Opa1, leading to a reduction in mitochondrial fusion.^73^ In addition, inhibition of drp1 can protect cells from cytotoxicity caused by mutated alpha‐synuclein.^74^ Studies have also suggested that alpha‐synuclein would readily bind to membrane and causes fragmentation of the membranes directly.^75^

One of the first key results to show that PD had a direct connection to mitochondria was the discovery of the mitophagy‐related proteins, Pink1 and Parkin, thus suggesting that mitophagy in particular is very important in PD. They discovered that some PD patient had mutations in these proteins. Mutations in Pink1^76^ and Parkin^77^ prevented normal function of mitophagy, as Pink1 was unable to phosphorylate OMM surface proteins, which prevented the clearance of damaged mitochondria. Pink1 has been found to interact with alpha‐synuclein itself in mitochondria and stimulate its clearance via mitophagy, reducing the toxicity caused by alpha‐synuclein. This effect was abolished with a mutated form of Pink1, the point mutation G309D, which prevented Pink1 from interacting with alpha‐synuclein and being cleared from the mitochondria.^78^

### 5.2 Huntington's disease

Huntington's disease (HD) is a devastating and fatal autosomal dominant inherited disease with no cure, causing motor dysfunction, memory deficits, and psychiatric symptoms*.* 79 It is characterized by mutations in the Huntington's gene (Htt), which are the result of abnormal expansion of the CAG repeats in the Htt gene, encoding an expanded polyglutamine (polyQ) stretch. Htt is then cleaved by proteolysis, which results in N‐terminus fragments, containing the expanded polyQ region. The Htt N‐terminus fragments become more prone to aggregation, and they accumulate together in the cytoplasm, locate especially in the striatum, as well as the frontal and temporal cortex. While the generation of cleaved mHtt is a common feature in HD, other forms of Htt are also thought to play a role in the progression of HD. Soluble, monomeric forms of mHtt, found separate from mHtt aggregates, were found to be the toxic species and directly contribute to the neurodegeneration seen in HD pathology.^80^, ^81^, ^82^, ^83^, ^84^

As with alpha‐synuclein and Aβ, Htt has been shown to bind directly to mitochondria. One studies showed that insoluble fragmented Htt could bind directly to Tim23.^85^, ^86^ Using Htt mutant primary cells, they discovered Htt prevents the import of important proteins into the mitochondria, and this could be reversed through overexpressing Tim23 protein.

When the aggregate involved accumulates in neurons (alpha‐synuclein in PD or Htt in HD), this can disrupt the function of the ETC Studies into HD patients have found reduced activity in complex II and complex III, as well as complex IV. A reduction in complex II in particular was discovered in the striatum of HD patients.^87^ In a transgenic mouse model of HD, they found that the structure of complex II was affected early in HD, and overexpressing complex II reduced the effects of mutated Htt on striatal neurons, relieving them from neuronal dysfunction. In other studies, this reduction in complex II was accompanied by a reduction in ATP production, as insufficient oxidative phosphorylation results in a disruption of mitochondrial metabolism.^86^, ^88^

The effect of mutant Htt on mitochondrial dynamics has also become a key focus in HD research. HD patients were found to have an imbalance in mitochondrial dynamics proteins, in postmortem tissue taken from the striatum of HD patients.^89^ They found decreased levels of fusion markers, such as Mfn1, Mfn2, and Opa1, and increased levels of fission markers drp1 and Fis1. This has subsequently been backed up with animal models and cells models. It was found that mutated Htt is able to interact with the mitochondrial fission GTPase protein drp1 and stimulate its activity directly. This caused mitochondria to fragment, leading to cellular dysregulation and death.^90^

### 5.3 Alzheimer's disease

In Alzheimer's disease (AD), the most common form of neurodegenerative dementia, the proteins amyloid beta, and tau form insoluble aggregates within the cell. AD pathology typically originates in the hippocampus and then develops into the neocortex, where damages in these regions can cause cognitive impairment such as memory decline and psychiatric disorder. The insoluble Aβ and tau aggregates accumulate over time, forming extracellular amyloid plaques, and intracellular neurofibrillary tangles, respectively, which disrupts normal mitochondrial function.^91^

Several studies have examined the effect of amyloid beta on mitochondrial function, with some reporting that that exposing amyloid beta to mitochondria can prevent protein import into the mitochondria, and this would result in a deterioration of the mitochondrial membrane potential.^92^ Studies have shown that AD patients showed reduced oxidative phosphorylation, accompanied by reduction in complex I, III, and IV.^93^ In a mouse model, expressing amyloid beta and tau, one study found complex I and complex IV were especially related to the dysregulation of oxidative phosphorylation, with complex I disrupted due to tau and complex IV due to amyloid beta.^94^, ^95^

As with PD, research into AD mitochondrial pathology determined that AD pathology was associated with changes in mitochondrial dynamics. Several papers have shown that both fusion and fission could be altered in AD. Although one paper has suggested that both fusion and fission markers are increased in AD,^96^ others report that mitochondrial fusion is decreased but mitochondrial fission is increased in AD, which as in HD and PD causes fragmentation of the mitochondria.^97^, ^98^ It has also been suggested that rather than drp1 causing mitochondrial fragmentation, the OMM receptor Fis1 levels were increased.^98^ These results do suggest that any imbalance in the mitochondrial dynamics may have a disastrous effect on mitochondrial function.

## 6 FINAL REMARKS

Mitochondria have a diverse role to play in cellular function and so there are several different mechanisms to protect the mitochondria from injury and disease. When these quality control mechanisms, such as mitophagy or mitochondrial dynamics, fail, there can be devastating effects, and therefore, further research into the mechanisms of how these control systems work, such as how exactly mitochondria fission occurs, will be very important.

## CONFLICT OF INTEREST

The authors declare no conflict of interest.
